# COVIDX-LwNet: A Lightweight Network Ensemble Model for the Detection of COVID-19 Based on Chest X-ray Images

**DOI:** 10.3390/s22218578

**Published:** 2022-11-07

**Authors:** Wei Wang, Shuxian Liu, Huan Xu, Le Deng

**Affiliations:** School of Information Science and Engineering, Xinjiang University, Urumqi 830017, China

**Keywords:** COVID-19, chest X-ray, coordinated attention, LSTM, lightweight CNN model

## Abstract

Recently, the COVID-19 pandemic coronavirus has put a lot of pressure on health systems around the world. One of the most common ways to detect COVID-19 is to use chest X-ray images, which have the advantage of being cheap and fast. However, in the early days of the COVID-19 outbreak, most studies applied pretrained convolutional neural network (CNN) models, and the features produced by the last convolutional layer were directly passed into the classification head. In this study, the proposed ensemble model consists of three lightweight networks, Xception, MobileNetV2 and NasNetMobile as three original feature extractors, and then three base classifiers are obtained by adding the coordinated attention module, LSTM and a new classification head to the original feature extractors. The classification results from the three base classifiers are then fused by a confidence fusion method. Three publicly available chest X-ray datasets for COVID-19 testing were considered, with ternary (COVID-19, normal and other pneumonia) and quaternary (COVID-19, normal) analyses performed on the first two datasets, bacterial pneumonia and viral pneumonia classification, and achieved high accuracy rates of 95.56% and 91.20%, respectively. The third dataset was used to compare the performance of the model compared to other models and the generalization ability on different datasets. We performed a thorough ablation study on the first dataset to understand the impact of each proposed component. Finally, we also performed visualizations. These saliency maps not only explain key prediction decisions of the model, but also help radiologists locate areas of infection. Through extensive experiments, it was finally found that the results obtained by the proposed method are comparable to the state-of-the-art methods.

## 1. Introduction

In December 2019, the COVID-19 virus was discovered and began to spread globally, in January 2020, the World Health Organization (WHO) declared it an “international public health emergency”, and by March 2020, the disease was considered a pandemic, in February 2022, the WHO prequalified its first monoclonal antibody to treat COVID-19, tocilizumab [1]. As of 16 October 2022, 621 million confirmed cases and 6.5 million deaths have been reported globally [2]. Although many countries are now actively developing and inoculating vaccines, since SARS-CoV-2 is an RNA virus, its biological characteristics determine that it is prone to mutation, and the long-term effectiveness of the vaccine cannot be guaranteed. Therefore, early detection of positive cases of COVID-19 disease and isolation of infected persons as soon as possible remain the mainstream response.

The standard diagnostic method for detecting coronavirus infection is reverse transcription polymerase chain reaction (RT-PCR), the results of which can be obtained within a few hours or two days, but this detection method is not only time-consuming and expensive [3], but the detection process also requires close contact between the doctor and the person to be tested. The diagnostic guidelines proposed by Wuhan Zhong nan Hospital indicate that COVID-19 can be assessed by detecting clinical symptoms and imaging manifestations of pneumonia [4].

Many researchers are also currently working on using modern computer science techniques to detect COVID-19 from lung images, such as deep learning [5,6,7,8,9], as in [5] the authors provide rapid analysis of chest radiology, biological samples, severity scores and other clinical data. Amir Hossein Barshooi et al. [6] addressed the data limitation problem by combining traditional data augmentation techniques with generative adversarial networks (GANs); while Bernheim et al. [7] concluded that the frequency of CT findings related to the time course of infection. Vinayakumar Ravi et al. [8] extracted features from the penultimate layer (global average pooling) of an EfficientNet-based pretrained model on CT images, and reduced the dimensions of the extracted features using kernel principal component analysis (PCA), then applied random forest and support vector machine (SVM) for prediction. Research by [9] summarized and reviewed some important research publications for DL-based classification of COVID-19 from CXR and CT images. As can be seen from these studies, CT and X-ray imaging systems are two effective tools for the initial, rapid identification and quantification of positive cases of COVID-19. Compared with the superior sensitivity of CT images, the disadvantages of CXR images are obvious: ordinary CXR images are the sum of the effects of X-rays on all tissues between the X-ray source and the capture sheet, and the tissue structure in X-rays is not very clear lacking 3D information [10]. However, the huge advantage of CXR is that it can be obtained cheaply and fast. Therefore, CXR is the better choice for initial evaluation.

The typical imaging findings of COVID-19 infection include ground-glass opacity (GGO) and airspace consolidation [11]. By analyzing chest X-ray images for observation, physicians can identify the initial stages of COVID-19 and the damage caused in the lungs, mainly in the lower lobes in later stages, as well as peripheral and subpleural distribution [12]. Diagnosing COVID-19 patients using this manual and traditional process is challenging because thousands of people are affected by COVID-19 every day, radiologists visit many patients each day and the diagnostic process takes a lot of time, so errors could significantly increase, and more false-negative results could occur which would take a huge toll on the patients and medical staff.

Although there are many challenges in manually understanding traces of COVID-19 infection from X-rays, subtle differences between COVID-19 and other types of X-rays can be traced through data patterns from convolutional neural networks (CNNs). Many recent studies on the diagnosis of COVID-19 disease from sample chest X-ray images have demonstrated the effectiveness of this approach in lung disease, and related studies [13] have found that COVID-like properties are located in the peripheral regions of the lung. In contrast, attributions for non-COVID pneumonia (NCP) occur in the middle of the lung. This is consistent with radiologists’ clinical observations of distinguishing COVID scans from NCP scans. Currently, many scholars are exploring ways to improve the results obtained by previous CNN methods and their limitations. However, most studies apply pretrained convolutional neural network (CNN) models and pass the features produced by the last convolutional layer directly into the classification head.

In this paper, our goal was to automatically and quickly analyze hidden features in X-ray images based on a CNN model for accurate classification of COVID-19, pneumonia (bacterial or viral pneumonia) and improve the sensitivity and specificity for COVID-19.

Therefore, in order to achieve the above goal, this paper selected lightweight networks such as Xception, MobileNetV2 and NasNetMobile as the original feature extractors, which have been pretrained on a large dataset called ImageNet and are widely used in image classification work. These three base feature extractors were then improved by using coordinated attention and LSTM. Finally, a fully connected layer, a dropout layer, and a softmax layer were added on top of the extracted features to build a new classification head. These improved base classifiers were used to classify chest X-ray images of patients. During testing, the base classifier was used to first generate prediction scores, and then these scores were combined using confidence fusion to generate the final class prediction. Finally, the whole model was named COVIDX-LwNet, and the classification process and results of a single base classifier were explained in detail using Grad-cam and visual feature maps.

Briefly, the main highlights of the current work are as follows:

- A novel ensemble model is proposed to combine the results of three deep learning-based classifiers by applying an ensemble method based on confidence fusion, a finely trained model based on Xception, MobileNetV2 and NasNetMobile.
- For the specific implementation of confidence fusion, a detailed description is given, the use of this ensemble approach helps reduce the chance of misclassification that can occur when relying on a single classifier.
- In addition to using a pretrained model as a feature extractor, each classifier also has a coordinated attention module and an LSTM layer.
- Feature map visualization was conducted on a single classifier to observe its feature learning process.

The rest of the paper is organized as follows. The second section introduces the research status in the field. Section 3 presents the dataset and proposed model. Section 4 presents experiments, including an ablation study on the model itself, the performance of the model for three- and four-class classification, as well as comparisons with other classical models or classification results from other state-of-the-art methods and generalization ability verification. Section 5 discusses the interpretability of the proposed model. Finally, Section 6 summarizes the full text and briefly discusses some of the limitations of this study and future research directions.

## 2. Basic and Background

The COVID-19 outbreak has seen numerous research efforts to develop deep learning-based diagnostic and screening methods using chest CXR imaging data. Image classification techniques have provided important results for diagnosis and prognosis in the medical field. According to the improvement of the model, it can be divided into four types:

(1) Transfer learning or fine-tuning of pretrained convolutional neural network models: For example, Soarov Chakraborty et al. [14] classified COVID-19, pneumonia, and healthy cases from chest X-ray images by applying a transfer learning method on a pretrained VGG-19 architecture. Ejaz Khan et al. [15] proposed EfficientNetB1 with a regularized classification head to detect chest X-ray classification of COVID-19. In this study, not only a deep learning model but also the hyperparameters were fine-tuned, thus significantly improving the performance of the fine-tuned deep learning model, in addition, the classification heads were regularized to improve performance.
(2) After the convolutional neural network model extracts features, machine learning, such as support vector machine or clustering, can be used as a classifier: such as Sourabh Singh Verma et al. [16]. In this paper, the support vector was combined in the last layer of the VGG16 convolutional network. For synchronization between VGG16 and SVM we added a layer of convolution, pooling and condensing between VGG16 and SVM, in addition, radial basis functions were used to transform and find the best results. Anupam Das [17] developed an ensemble learning based on CNN deep features (ELCNN-DF), in which the deep features are extracted from the pooling layer of the CNN, the fully connected layer of CNN is called “Support Vector Machine” for three classification device replacement. SVM, Autoencoders, Naive Bayes (NB), the final detection of COVID-19 is performed by these classifiers, where a high-ranking strategy is used.
(3) Integrated multiple convolutional neural network models: For example, Anubhav Sharma et al. [18] proposed a method that identified patients infected with SARS-CoV-2 from chest X-ray images of healthy and/or pneumonia patients: COVDC-Net, which uses two modified pretrained models (on ImageNet), MobileNetV2 and VGG16, respectively, and removes the classifier layer and fuses the two models using a confidence fusion method. Jingyao Liu et al. [19] proposed an effective method based on deep learning, deep feature fusion classification network (DFFCNet), to improve the overall diagnostic accuracy of diseases. The method is divided into two modules, deep feature fusion module (DFFM) and multiple disease classification module (MDCM). DFFM combines the advantages of different networks (EfficientNetV2 and ResNet101) for feature fusion, and MDCM uses support vector machine (SVM) as a classifier to improve the classification performance.
(4) Development of a new convolutional neural network model: Saddam Hussain Khan et al. [20] developed a new CNN architecture STM-RENet to explain the radiographic patterns of X-ray images, and the authors proposed a new convolutional Block STM, which can implement region- and edge-based operations separately or jointly. The use of a system that combines region and edge realization with convolution operations facilitates exploration of region homogeneity, intensity inhomogeneity, and boundary-defining features. Additionally, exploiting the idea of channel boosting by using transfer learning to generate auxiliary channels from two additional CNNs, which are then connected to the original channels of the proposed STM-RENet, developed CB-STM-RENet, which utilizes channel boosting and learning texture changes to effectively screen X-ray images for COVID -19 infection further enhances the learning ability of STM-RENet. Md. Kawsher Mahbub et al. [21] developed a lightweight deep neural network (DNN) for unhealthy CXR screening with reduced epoch and parameters.

The following conclusions can be drawn from the above studies: At the beginning of the COVID-19 outbreak, due to the lack of samples, many researchers proposed many models suitable for small amounts of data from this perspective. Some researchers use transfer learning for training because it allowed the network to have a higher initial performance, faster training speed, and a better convergence of the resulting model, which can reduce network parameters and make the network smaller. However, in the process of learning disease features, there are huge differences between medical images and natural images. Often the scene is single and the structure is fixed. Deep learning usually pays attention to some features that are not related to the disease, which leads to a poor generalization ability of the obtained model. Most of the current research on COVID-19 use a single CNN network for learning, and there is a problem of incomplete or useless feature extraction. Different networks extract different features. Therefore, it is very necessary to develop a new method that combines multiple CNN networks for learning.

Base classifiers in this paper preferentially selected the pre-trained models of Xception, MobileNetV2 and NasNetMobile, which are lightweight networks, and introduced coordinated attention, LSTM and a new classification head as improvements. The outputs were integrated using confidence fusion to further improve detection accuracy.

## 3. Materials and Methods

In this section, the datasets used for the training and testing are discussed along with the deep learning models used in this study. The datasets are further discussed in Section 3.1. Likewise, the proposed methodology for the classification of COVID-19 infection is discussed in Section 3.2.

### 3.1. Dataset

To test our proposed method, we collected three datasets from different public sources: Dataset-1 (D1), Dataset-2 (D2) and Dataset-3 (D3).

D1 used the COVID19-pneumonia-normal-chest-xray-pa-dataset dataset available to the public on Kaggle. It consisted of images collected from GitHub repositories, Kaggle, Radiopedia, the Italian Society of Radiology (SIRM) and the Figshare data repository website. D2 adopted the CoroNet dataset from the study by Asif Iqbal Khan et al. [22]. It provided COVID-19 X-ray images in the open source Github repository by Joseph et al. The pneumonia bacteria, pneumonia virus, and normal chest X-ray images were all from the Kaggle repository “Chest X-ray Images (Pneumonia)”. D3 used a curated dataset of post-COVID-19 anterior chest radiographic images (X-rays) published by the Indian Institute of Science, PES University, MS Ramaiah Institute of Technology, Concordia University.

D1, D2 and D3 are shown in Table 1. In all experiments in this paper, the split ratio of training set and test set was 8:2.

### 3.2. Proposed Methodolgy

**Figure 1:**
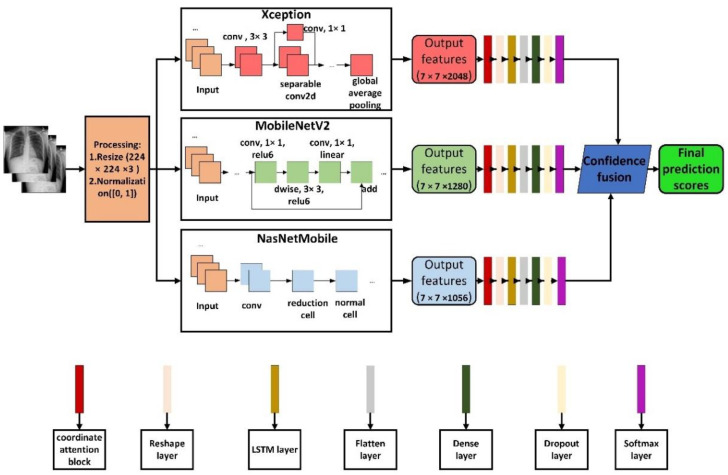
Specific details of the proposed architecture.

In this work, the architecture of the whole method is shown in Figure 1, which was a lightweight network ensemble model for detecting COVID-19 based on chest X-ray images, named COVIDX-LwNet in this paper. In other subsections of this section, the basic concepts used in this work, such as the pretrained CNN models, coordinated attention, LSTM, and confidence fusion methods are discussed. In addition, the hardware and hyperparameter settings for the experiments of the proposed method are also described.

As shown in Figure 1, first, the 224 × 224 × 3 chest X-ray images are normalized and fed into the base classifier. Pretrained models, Xception, MobileNetV2 and NasNetMobile extract feature maps of size 7 × 7 × 2048, 7 × 7 × 1280 and 7 × 7 × 1056, respectively.

Next, these feature maps are passed through a coordinated attention module to decompose the channel attention into two one-dimensional feature encoding processes that aggregates features along two spatial directions, respectively. Then, the resulting feature maps are encoded as a pair of direction-aware pairs and a position-sensitive attention map, which can be complementary applied to the input feature map to increase the representation of objects of interest.

After this, the feature map from the coordinated attention module is fed into the Reshape layer, which changes the shape from (h, w, c) to (h * w, c) for input into the LSTM.

LSTM enhances the performance of the model by maintaining the state information of the features encountered in the previous generation of image classification and speed up the convergence.

Finally, the features extracted by LSTM are inputted into the reconstructed classification head, namely a Flatten layer, a Dense layer with 512 units, a Dropout layer with a dropout rate of 0.5, and an output unit of 3 (in the four classifications, only the output unit of the Softmax layer is changed to 4, and the rest are not changed).

During the testing process, the test samples are first passed through three base classifiers to obtain the corresponding predicted scores, which are then used to confidently fuse the obtained outputs to obtain the final predicted labels.

Three base classifiers and the proposed model (COVIDX-LwNet) were all implemented in Keras based on Tensorflow 2.5. The original models of the three base classifiers were all pretrained on the ImageNet dataset. After improvement, 10% of the data was first divided from the training set as the validation set, and then the Adam optimizer was used on the prepared dataset. End-to-end retraining was performed with a learning rate of 2 × 10^−4^, a batch size of 32, and an epoch value of 50. For training, data shuffling was enabled, which involved shuffling the data before each epoch. All experiments and training were performed on a Linux server equipped with a 3090 graphics card, and the CPU model AMD EPYC 7601, 16 cores, 64 G.

#### 3.2.1. Pretrained CNN Model

The layer weights of large CNN models (like AlexNet, DenseNet, etc.) are frozen after being trained on large datasets, such as ImageNet. Therefore, researchers with low computing equipment and small datasets can remove the last classification layer of the pretrained model, add their own custom layers, and use the other layers described above as feature extractors.

In applications such as COVID-19 detection, the differences in chest X-ray images from different categories are less pronounced. In the current work, a CNN model was used as a feature extractor, and the main role of the CNN was to convert the image as input into a form that is easy to analyze without losing any useful information needed for prediction.

Furthermore, the limited amount of available data discourages the use of large models: in fact, training smaller models is a safer option, as they are less prone to overfitting the data. Very large models, such as DenseNet-121, without proper regularization, tend to memorize the entire dataset, negatively impacting the generalization ability [23]. Therefore, when we chose the model, we tried our best to consider the lightweight and simple model, and the model with better initial classification effect through simple experiments was used as the feature extractor.

The CNN model used here is described below:

- Xception: Xception [24] is another improvement of Inception-v3 proposed by Google after Inception. On the basis of Inception v3, the Inception module is replaced with a depth-wise separable convolution, and then combined with the skip connection of ResNet, Proposed Xception.
- MobileNetV2: MobileNetV2 [25] extends feature extraction and introduces an inverted residual structure. The model architecture consists of a convolutional layer and a series of residual bottleneck layers. The kernel size for all spatial convolution operations adopts ReLU6 as nonlinearity, as well as batch normalization and dropout in the training phase. Each bottleneck block consists of 3 layers, starting with a (1 × 1) convolutional layer, followed by the aforementioned (3 × 3) depth-wise convolutional layer, and finally another (1 × 1) volume without ReLU6 activations laminate. MobileNetV2 is currently widely used due to its excellent feature extraction capability and small size.
- NasNetMobile: NasNet [26] is a scalable CNN architecture (built through neural architecture search) consisting of basic building blocks (units) optimized using reinforcement learning. A unit consists of only a few operations (several separable convolutions and pooling) and is repeated as many times as the network requires. The mobile version (NasNetMobile) consists of 12 cells.

#### 3.2.2. Coordinated Attention

**Figure 2:**
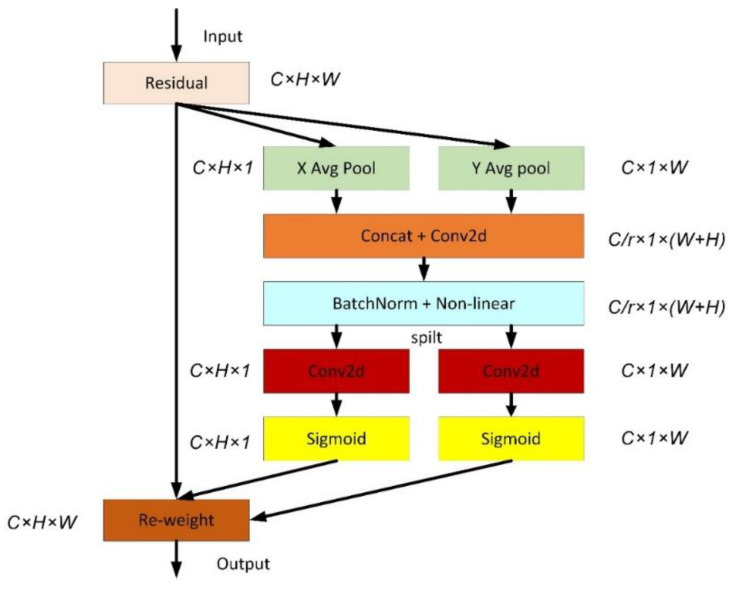
Coordinated Attention.

Coordinated attention is a new mobile network attention mechanism proposed by Qibin Hou et al. [27], which embeds location information into channel attention, called “coordinated attention”, and its structure is shown in Figure 2.

Unlike channel attention, which converts a feature tensor into a single feature vector through 2D global pooling, coordinate attention decomposes channel attention into two 1D feature encoding processes that aggregates features along two spatial directions, respectively. This approach can capture long-range dependencies in one spatial direction while preserving precise location information in the other. The resulting feature maps are then encoded as a pair of orientation-aware and position-sensitive attention maps, respectively, which can be complementary applied to the input feature maps to augment the representation of objects of interest. This coordinate concern is simple and can be flexibly plugged into the network with little computational overhead.

#### 3.2.3. Long Short-Term Memory (LSTM)

**Figure 3:**
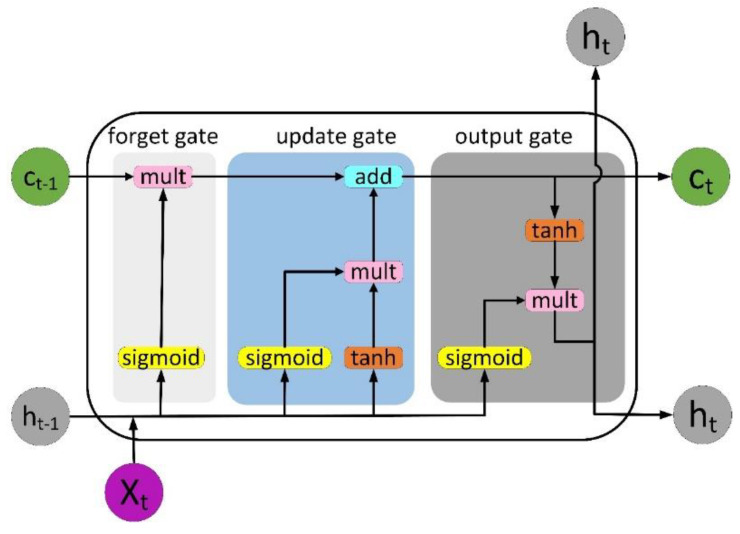
The internal structure of LSTM.

Long short-term memory is an improvement over recurrent neural networks (RNNs) [28]. LSTM proposes memory blocks instead of traditional RNN units when addressing vanishing and exploding gradients. It then adds a cell state to hold the long-term state, which is its main difference from an RNN. LSTM networks can remember previous information and connect it to the currently acquired data. LSTM combines three gates: input gate, forgetting gate and output gate. The value range of the gate is (0,1), controlled by the sigmoid function, where x_t_ refers to the current input, C_t_ and C_t-1_, respectively, represent the new and previous cell states, h_t_ and h_t-1_ are the current and previous outputs, respectively. The internal structure of LSTM is shown in Figure 3.

#### 3.2.4. Confidence Fusion

**Figure 4:**
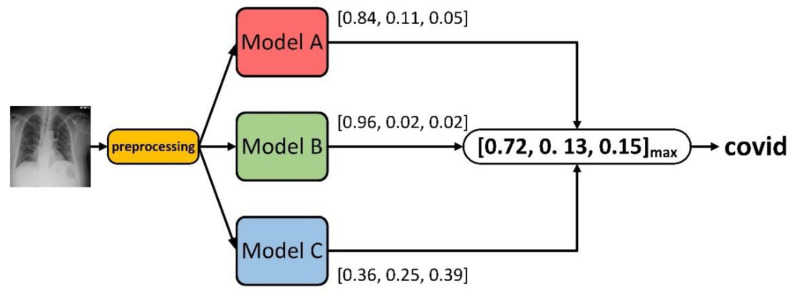
Schematic diagram of confidence fusion.

Different layers of a convolutional neural network give different outputs. The output from multiple models at different stages can be used to generate combined classification results, which can often provide better results than the parent model. One such method is to fuse the output vector [29] (classification probability vector) from the last Softmax classifier layer, e.g., confidence fusion, the process of which is schematically shown in Figure 4. Let p_i_ be the output of a given CNN model and denoted as (p_i,1_, p_i,2_, ........., p_i,n_), where i denotes the CNN under consideration and n denotes the classification number class, where I is the total number of models fused. The term p_i,j_ represents the probability that the i-th CNN classifies the given sample as the j-th class. The mean value for each category is calculated according to Equation (1):

(1) $$\overset{-}{p_{j}}=\sum_{i=1}^{I}p_{i,\;j}$$

Among the n classes, the final predicted class c is given by Equation (2).

(2) $$c=\mathrm{argmax}\;\overset{-}{\mathrm{pj}}\;\mathrm{where}\;1<\;j<\;n$$

## 4. Experiments

### 4.1. Performance Parameters

Once the model was trained, performance parameters based on the confusion matrix were computed from each stage of the proposed method. The performance parameters, namely, Precision, Sensitivity (Recall), Specificity, F1 and Accuracy are expressed as:

(3) $$\mathrm{Precision}=\frac{\mathrm{TP}}{\mathrm{TP}+\mathrm{FP}}$$

(4) $$\mathrm{Sensitivity}\left(\mathrm{Recall}\right)=\frac{\mathrm{TP}}{\mathrm{TP}+\mathrm{FN}}$$

(5) $$\mathrm{Specificity}=\frac{\mathrm{TN}}{\mathrm{TN}+\mathrm{FP}}$$

(6) $$F1=2\times \frac{\mathrm{Precision}\times \mathrm{Recall}}{\mathrm{Precision}+\mathrm{Recall}}$$

(7) $$\mathrm{Accuracy}=\frac{\mathrm{TP}+\mathrm{TN}}{\mathrm{TP}+\mathrm{FN}+\mathrm{TN}+\mathrm{FP}}$$

TP, TN, FP and FN are confusion matrix elements (see Table 2).

### 4.2. Three Classifications (Dataset D1)

In this subsection, the performance of the different components of the proposed model and the performance of tri-classification are discussed, for which many experiments were performed on the described dataset.

However, for simplicity, dataset D1 was dedicated to ablation experiments and tri-classification (COVID-19, normal and other pneumonia) experiments. The final variant (i.e., after adding a coordinated attention module and an LSTM layer and a new classification head) was considered here as a base classifier and was used to design an ensemble method based on confidence fusion. Now, Xception + Coordinated Attention module + LSTM layer + new classification head, MobileNetV2 + Coordinated Attention module + LSTM layer + new classification head, and NasNetMobile + Coordinated Attention module + LSTM layer + new classification head are called Base Classifier 1, Base Classifier 2, and Base Classifier 3, respectively.

First, there were two groups of ablation experiments. The purpose of the first set of ablation experiments was to verify whether the added components can improve the performance of existing classifiers. The purpose of the second set of ablation experiments was to verify how much removing a certain component affects the improved classifier. The results of the two sets of experiments are presented in Table 3 and Table 4, respectively.

From the results of the first group of ablation experiments in Table 3, it can be seen that compared with the original CNN, the addition of CoordAtt and new classification header improved the classification performance of the original CNN, especially the performance improvement brought by CoordAtt is the most obvious. The LSTM only showed a slight improvement on the original models of Xception, MobileNetV2 and NASNetMobile, with an increase of 0.15%, 0.07% and 0.05%, respectively. According to the results of the second set of ablation experiments in Table 4, it can be seen that after removing the CoordAtt or new classification header, the performance of the specific basic classifier was negatively affected. Among them, the basic classifier 2 (MobileNetV2) was most affected by CoordAtt. The most affected by the new classification header was the basic classifier 3 (NASNetMobile), which was reduced by 0.46%. Therefore, it is safe to say that using the coordinated attention module and the new classification header helped to improve the performance of the base classifier, and the addition of LSTM had a slight improvement.

The base classifier and confidence fusion-based ensemble methods also recorded precision, recall, F1 score, and test accuracy, which were given by averaging the results of 5-fold cross-validation. Table 5 shows the performance of the final proposed architecture, COVIDX-LwNet, at each compromise.

**Figure 5:**
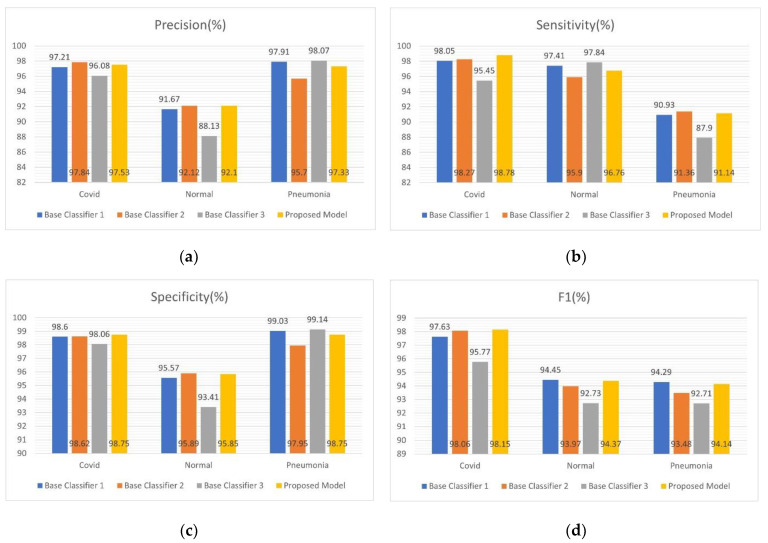
Three base classifiers are shown (base classifier 1 in the figure: Xception + coordinated attention module + LSTM layer + new classification head, base classifier 2: MobileNetV2 + coordinated attention module + LSTM layer + new classification head, base classifier 3: NasNetMobile + Coordinated attention module + LSTM layer + new classification head) and the proposed ensemble model COVIDX-LwNet (the model proposed in the figure) based on confidence fusion on the test set of the D1 dataset. In (**a**) precision, (**b**) sensitivity, (**c**) Specificity, (**d**) F1 score of all three base classifiers and the proposed ensemble method are shown, respectively.

**Figure 6:**
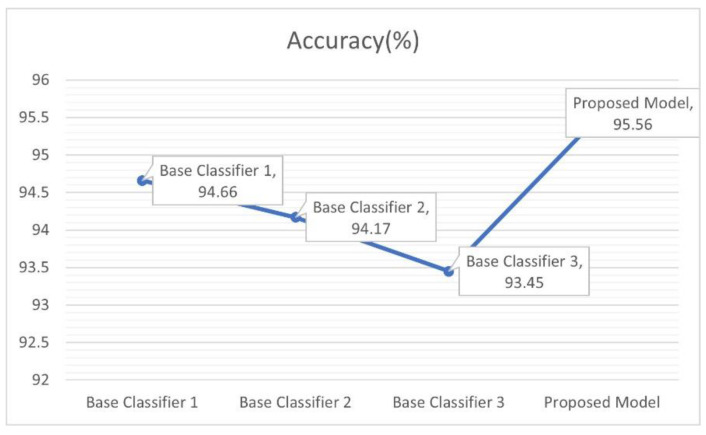
The accuracy of the above four models.

Figure 5a,d show the accuracy, sensitivity, specificity and F1 score of all three base classifiers and the proposed ensemble method, respectively. It is worth mentioning that, the sensitivity and specificity of the proposed model COVIDX-LwNet in the COVID category were both above 98.7%, respectively, which indicates that the proposed model (COVIDX-LwNet) can be more effective in reducing the false negative rate of new coronary pneumonia. While Figure 6 records the accuracy of testing each of the above models, the ensemble method based on confidence fusion was superior to all basic classifiers in accuracy, reaching the highest 95.56% among the four models.

**Figure 7:**
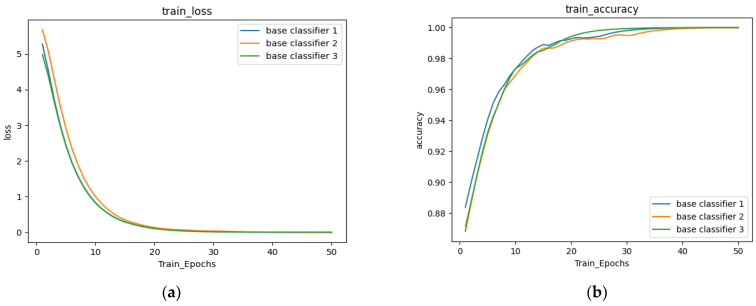

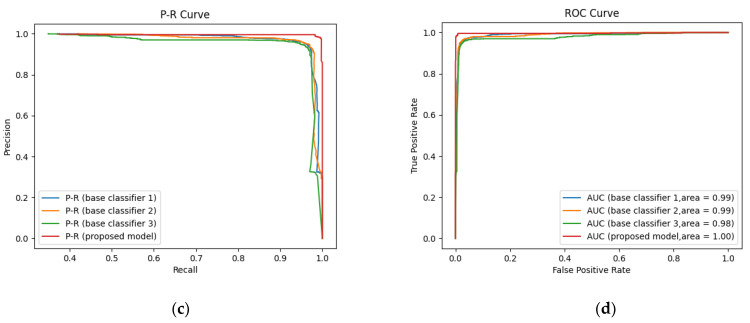
Three-class correlation curves for the three base classifiers and the proposed model (C ovidX-LwNet). (**a**) train loss, (**b**) train accuracy, (**c**) P-R, (**d**) ROC.

The training properties of the training accuracy curves and training loss curves of the three base classifiers are also shown in Figure 7a,b, and their trends with the proposed model’s P- R and ROC curves are shown in as can be seen from these curves in Figure 7c,d, overall, the proposed model was well trained to handle the current three classes of problems.

### 4.3. Four Classifications (Dataset D2)

In this section, the performance of the proposed model (COVIDX-LwNet) on four classifications (COVID-19, normal, bacterial pneumonia, and viral pneumonia) on dataset D2 will be discussed in detail, as well as comparison of the results with other models. The COVIDX-LwNet applied to the four-class is a modification of the three-class COVIDX-LwNet described in Section 4.1. There were three main changes: first, the output unit of the last layer of softmax layer was changed to four; secondly, considering the size of the data set D2, the batch size was changed to 16; finally, the learning rate was changed from 2 × 10^−4^. If it remains unchanged for three epochs, it decays to 0.5 times the original learning rate. The rest were consistent with the experimental setup in Section 3.2.

**Figure 8:**
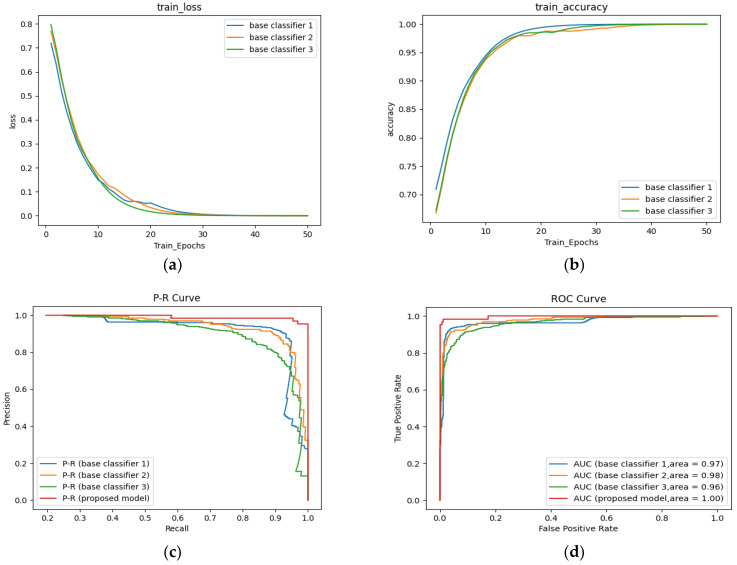
Four-class correlation curves for the three base classifiers and the proposed model (COVIDX-LwNet). (**a**) train loss, (**b**) train accuracy, (**c**) P-R, (**d**) ROC.

The base classifier and confidence fusion-based ensemble methods also recorded precision, sensitivity, F1-score, and test accuracy, which were given by averaging the results of 5-fold cross-validation. Table 6 shows the performance of the final proposed architecture, COVIDX-LwNet at each compromise, the final average test accuracy was 91.2%. The training properties of the training accuracy curves and training loss curves of the three base classifiers are also shown in Figure 8a,b, and their trends with the proposed model’s P-R and ROC curves are shown in Figure 8c,d. Overall, the proposed model is well-trained and performed well on the current four classes of problems.

In addition, we also condudcte a detailed comparison of the proposed model COVIDX-LwNet with those proposed in two other papers on the D2 dataset.

Wang and Wong [31] proposed a residual deep architecture called COVID-Net for detecting COVID-19 from chest X-ray images. COVID-Net, one of the early works on COVID-19, used deep neural networks to classify chest X-ray images into four categories (COVID, normal, pneumonia bacteria, and pneumonia virus), and COVID-Net achieved 83.5 in four categories % accuracy.

Asif Iqbal Khan et al. [22] proposed CoroNet, a deep convolutional neural network model for automatic detection of COVID-19 infection from chest X-ray images. The overall accuracy rate on pneumonia bacteria and pneumonia virus) reached 89.6%, of which the accuracy and recall rate of the COVID-19 case were 93.2% and 98.2%, respectively. It should be noted that the D2 dataset used in this paper is also derived from this study.

Table 7 shows that COVID-Net, performance comparison of CoroNet and our proposed model (COVIDX-LwNet) on dataset D2 on a four-class classification task.

### 4.4. Comparison (Dataset D3)

We also compared the performance of our system with other deep learning methods on dataset D3. Due to the obvious imbalance of the D3 dataset, in order to facilitate us to perform, three-class and four-class on the D3 dataset, two different datasets ((D3_1 to D3_2) generated from the D3 dataset are listed below) combinations.

D 3_1 (three categories): In the D3 dataset, all the 1281 sheets of the COVID category, the first 1281 sheets of the normal category, the first 641 sheets of the pneumonia_bacteria category and the top 641 sheets of the pneumonia_viral category together form pneumonia. The class was 1282 sheets.

D3_2 (four classifications): In the D3 dataset, all the 1281 sheets of the COVID category, the first 1281 sheets of the normal category, the first 1281 sheets of the pneumonia_bacteria category and the first 1281 sheets of the pneumonia_viral category.

The experimental setup remains the same as in Section 3.2.

First, we applied popular pretrained networks on dataset D3: VGG16, ResNet50, and DenseNet121, as well as base classifier 1 (Xception), base classifier 2 (MobileNetV2), and the proposed model (COVIDX-LwNet) in this paper. The category recognition was performed using the Softmax layer of the pretrained network. We first compared two of the most commonly used indicators to measure the amount of model complexity and the amount of calculation, the parameters and FlOPs of the model are in Table 8.

Second, on the D3 dataset specifically used for testing and comparison, we used a five-fold cross-validation method to train the model. In the testing phase, the three-classification of the model adopted the test accuracy obtained from the D3_1 dataset training and D1 dataset testing, which was the same as the test accuracy obtained from the D3_1 dataset training and D3_1 dataset testing (that is, the model was trained and tested on the same dataset). We then rated and compared the differences between the above two to evaluate the generalization ability of the model. The model’s four-class generalization ability verification (on the D3_2 and D2 datasets) was the same as the three-class.

Therefore, in the comparative experiments in this subsection, we conducted four sets of experiments: the first set of D3_1:D3_1 (e.g., training set: test set), the second set of D3_1:D1, the third set of D3_2:D3_2, and the fourth set of D3_2:D2. The first two groups were three-category experiments, and the latter two groups were four-category experiments.

Table 9, Table 10, Table 11 and Table 12, show the accuracy of each model on different tasks. Compared with other traditional pretrained models, the proposed model achieved 99.15% and 94.86% high accuracy in the first and third sets, respectively, and showed superior performance in the second and fourth sets of experiments.

Furthermore, our method results are promising and comparable to other deep learning studies for COVID-19 detection. Other research results on DL and the details of their respective datasets are shown in Table 13.

## 5. Visualization

Visual analysis of neural networks is of great significance in both research and practical applications. Gradient-weighted class activation map (Grad-CAM) [37] analysis helps us understand the inner workings of CNN models. It does this by using a heatmap that shows important regions of the input image according to the model. Through this, it is possible to verify that the convolutional model and highlights regions of interest that are required for the classification task.

**Figure 9:**
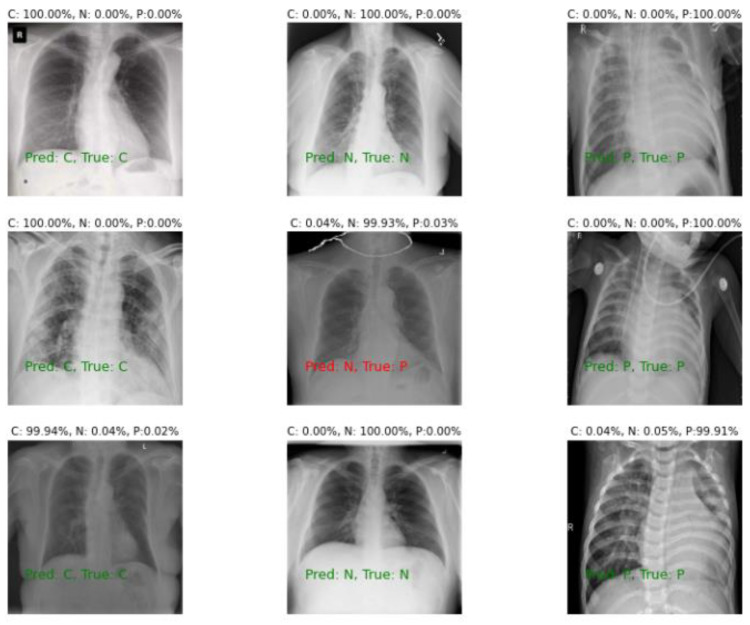
Three classification results of some sample images of the test set in dataset D1 (three random images for each category, nine images in total), the true label in the leftmost column is COVID, and the true label in the middle column is normal, the ground truth label for the rightmost column is pneumonia.

Taking our improved Xception model (e. g. base classifier 1) as an example, Figure 9 shows the three-classification results of some sample images of the test set in dataset D1 three images are randomly selected for each category, for a total of nine images. The green font means the predicted class is consistent with the real class, and the prediction error is the red font. It was found through Figure 9 that the predicted classes had much higher values than the undetected classes, which indicates that the proposed COVIDX-LwNet method accurately extracted disease-related features, and these features were only matched to specific classes. However, we still found that despite the excellent overall accuracy of the COVIDX-LwNet method, its predicted wrong class gave a probability of about 99%. Literature [38] have already discussed how deep neural networks can be easily fooled and produce high-confidence predictions based on junk images, so this may be a serious weakness of CNNs in general. CNNs are not without errors, even if they have high accuracy. Especially in the medical field where critical decisions are made, it is crucial to understand the errors associated with predictions, which can be further addressed in future studies.

**Figure 10:**
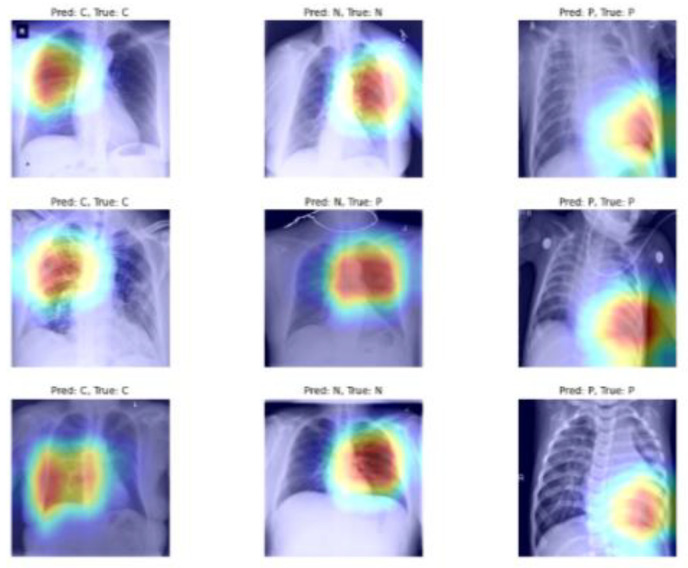
Raw chest X-ray image corresponding to Figure 9 and Grad-CAM visualization generated using the following model: Xception model + coordinated attention module + LSTM layer + new classification head.

Figure 10 shows the Grad-CAM analysis of the nine images above. As can be seen from Figure 9 raw X-ray images, they contain unwanted text and other symbols that may affect the performance of the classifier, but from the Grad-CAM visualization, it is important to note that the model does not focus on unwanted information and only looks at the areas that actually help with the classification task. Therefore, in Figure 10, it can be seen that the COVID-19 chest X-ray image was not clear, but the classifier still manages to classify it by focusing on important regions of the lungs.

**Figure 11:**
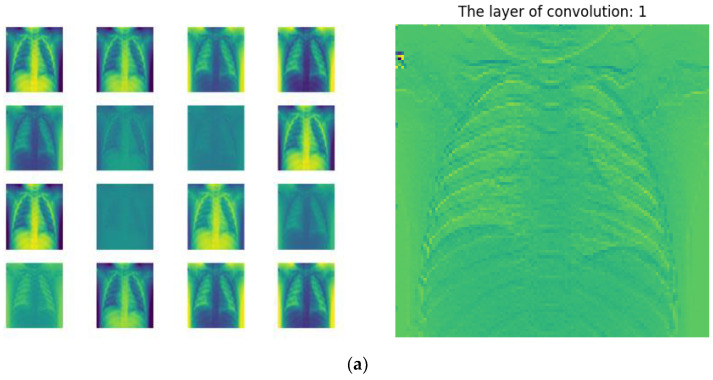

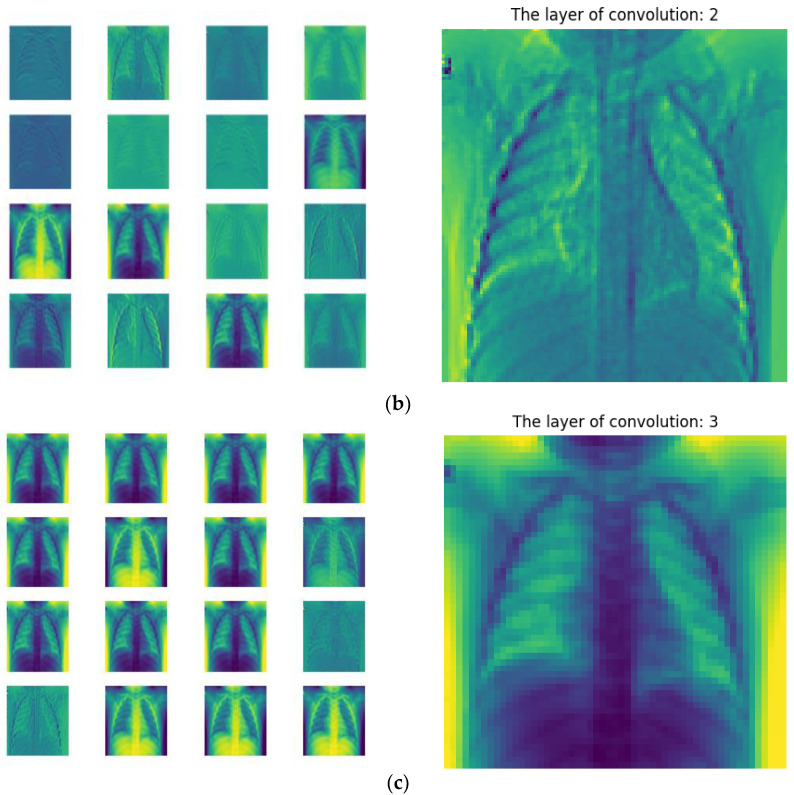
(**a**–**c**) shows the first 16 channel maps of the features extracted by the first three convolutional layers of base classifier 1 and their overlay fusion on the right, some feature maps are clearly specialized to detect areas where the thorax and lungs are present, others highlight only areas with thickened lung markings, pulmonary fibrosis and bilateral diffuse opacities.

In addition, visualizing the intermediate feature map refers to the feature map showing the output of each convolutional and pooling layer in the network for a given input (the output of a layer is often referred to as the activation of the layer, that is, the output of the activation function). This allows us to see how the input is decomposed into the different filters that the network learns. We wished to visualize feature maps in three dimensions: width, height and depth (channels). Each channel corresponds to a relatively independent feature, so the correct way to visualize these feature maps is to plot the content of each channel as a two-dimensional image. Figure 11 shows an example of features learned by base classifier 1 on CXR images in the first 16 channel maps of features extracted from the first, second and third convolutional layers and their overlay fusion.

## 6. Conclusions

The application of pattern recognition techniques has proven to be very useful in many real-world situations. Several papers [10,13,39,40,41] proposed the use deep learning methods to identify pneumonia and COVID-19 in CXR images with encouraging results. Therefore, given the recent trend of using deep learning models for early detection of COVID-19 cases, this paper proposed a method for COVID-19 detection using chest X-ray images as input data, named COVIDX-LwNet. The proposed method uses a confidence fusion to combine the outputs of three base classifiers—Xception, MobileNetV2, and NasNetMobile. Prior to this, the performance of the feature extractor performance was enhanced by applying coordinated attention module, a LSTM layer, and a new classification header to form an improved base classifier. After various experiments, the results demonstrate that the ensemble classification model based on confidence fusion significantly improves the performance of a single classification model. Furthermore, this ensemble model is comparable to state-of-the-art models in both three-class and four-class cases.

Although, COVIDX-LwNet performed well when compared to other state-of-the-art COVID-19 detection methods, there is still room for improvement. First of all, in experiments we found that the addition of LSTM only slightly improved the final results of the three base classifiers, but the main purpose of using LSTM was to enhance the model by maintaining the state information of the features encountered in the previous generation of image classifications. This feature can significantly speed up the convergence of the model. This finding prompted us to retain the addition of LSTM, and the future research goal is to find or construct similar functional but more efficient components to replace LSTM.

Second, it can be seen that the proposed method misclassified some test data samples, and the reason for this misclassification can be attributed to the performance of the individual classifiers. This can be improved by using a more powerful set of classifiers than the current one and by increasing the number of combined classifiers using confidence fusion methods. In addition to that, its predicted wrong class gave a probability of about 99%, so incorporating uncertainty [42] into the predictions and providing error bars for the CNN predictions to allow medical staff to manually review mispredictions may be a challenge for current models. 

Finally, it is planned to use this method for other medical image classification tasks in the future.

## Figures and Tables

**Table 1:** Specific composition of D1, D2 and D3.

Dadaset	Source Address	Class (Number of Samples)	Total
**D1**	www.kaggle.com/amanullahasraf/COVID19-pneumonia-normal-chest-xray-pa-dataset (accessed on 17 June 2022)	COVID (2313)	6939 images
		normal (2313)	
		pneumonia (2313)	
**D2**	https://github.com/drkhan107/CoroNet (accessed on 18 August 2022)	COVID (320)	1638 images
		normal (445)	
		pneumonia_bacteria (449)	
		pneumonia_viral (424)	
**D3**	https://data.mendeley.com/datasets/9xkhgts2s6/1 (accessed on 18 August 2022)	COVID (1281)	9208 images
		normal (3270)	
		pneumonia_bacteria (3001)	
		pneumonia_viral (1656)	

**Table 2:** Correlation calculations of confusion matrix (Cited from literature [30]).

Based on the Gold Standard			
	Disease Present	Disease Absent	Total
Predicted Model Positive	True positive (TP)	False positive (FP)	TP + FP
Predicted Model Negative	False negative (FN)	True negative (TN)	FN + TN
Total	TP + FN	FP + TN	TP + FP + FN + TN

**Table 3:** The first set of ablation experiments, evaluation metric: Accuracy (%).

	Xception	MobileNetV2	NASNetMobile
**CNN**	93.32	91.87	92.88
CNN + CoordAtt *	94.12	93.96	93.10
**CNN + CoordAtt + LSTM**	94.27	94.03	93.15
**CNN + CoordAtt + LSTM + New classification header**	94.66	94.17	93.45

* CoordAtt means the abbreviation of Coordinated Attention.

**Table 4:** The second set of ablation experiments, evaluation metric: Accuracy (%).

		Xception	MobileNetV2	NASNetMobile
**CoordAtt**	w/o *	94.18	91.97	92.88
**LSTM**	w/o	94.45	93.88	93.38
**New classification header**	w/o	94.27	94.03	93.15
**CoordAtt + LSTM + New classification header**	w/ * + w/ + w/	94.66	94.17	93.45

* w/o means the abbreviation of without, w/ means the abbreviation of with.

**Table 5:** The performance of the final proposed architecture, COVIDX-LwNet, at each compromise (D1, 3 class).

Class	Metric	Fold					
		1st Fold	2nd Fold	3rd Fold	4th Fold	5th Fold	Average
COVID	Precision	98.49	97.24	95.57	97.65	98.70	97.53
	Sensitivity	98.92	99.13	98.05	99.13	98.70	98.78
	Specificity	99.24	98.60	97.73	98.81	99.35	98.75
	F1	98.70	98.18	96.79	98.39	98.70	98.15
Normal	Precision	92.68	91.84	91.58	92.42	91.99	92.10
	Sensitivity	98.49	94.82	96.33	97.41	96.76	96.76
	Specificity	96.11	95.78	95.57	96.00	95.78	95.85
	F1	95.50	93.30	93.89	94.85	94.32	94.37
Pneumonia	Precision	98.61	95.90	97.89	98.14	96.13	97.33
	Sensitivity	92.01	90.93	90.28	91.36	91.14	91.14
	Specificity	99.35	98.05	99.03	99.14	98.16	98.75
	F1	95.20	93.35	93.93	94.63	93.57	94.14
	Accuracy	96.47	94.96	94.88	95.97	95.53	95.56

**Table 6:** The performance of the final proposed architecture, COVIDX-LwNet, at each compromise (D2, 4 class).

Class	Metric	Fold					
		1st Fold	2nd Fold	3rd Fold	4th Fold	5th Fold	Average
**COVID**	Precision	100	98.39	100	96.83	100	99.04
	Sensitivity	95.31	95.31	96.88	95.31	96.88	95.94
	Specificity	100	99.62	100	99.24	100	99.77
	F1	97.60	96.83	98.41	96.06	98.41	97.46
**Normal**	Precision	90.53	92.31	93.62	92.55	92.55	92.31
	Sensitivity	96.63	94.38	98.88	97.75	97.75	97.08
	Specificity	96.23	97.07	97.49	97.07	97.36	97.08
	F1	93.48	93.33	96.17	95.08	95.08	94.63
**Pneumonia_Bacteria**	Precision	88.76	86.17	92.31	85.54	93.98	89.32
	Sensitivity	87.78	90.00	80.00	78.89	86.67	84.67
	Specificity	95.80	94.54	97.48	94.96	97.90	96.14
	F1	88.27	88.04	85.71	82.08	90.17	86.85
**Pneumonia_Viral**	Precision	87.95	88.89	81.91	79.55	86.52	84.96
	Sensitivity	85.88	84.71	90.59	82.35	90.59	86.82
	Specificity	95.88	96.30	93.00	92.59	95.06	94.57
	F1	86.90	86.75	86.03	80.92	88.51	85.82
	Accuracy	91.16	90.85	91.20	90.11	92.68	91.20

**Table 7:** COVID-Net, four-class performance comparison between CoroNet and COVIDX-LwNet.

Class	COVID-Net			CoroNet			COVIDX-LwNet (Ours)		
	Prec. (%) *	Sen. (%)	F1 (%)	Prec. (%)	Sen. (%)	F1 (%)	Prec. (%)	Sen. (%)	F1 (%)
**COVID**	80	**100**	88.8	93.17	98.25	95.61	**99.04**	95.94	**97.46**
**Normal**	95.1	73.9	83.17	**95.25**	93.5	94.3	92.31	**97.08**	**94.63**
**Pneumonia_Bacteria**	87.1	**93.1**	**90**	86.85	85.9	86.3	**89.32**	84.67	86.85
**Pneumonia_Viral**	67.0	81.9	73.7	84.1	82.1	83.1	**84.96**	**86.82**	**85.82**
**# of Parameters**	**116 million**			33 million			71 million		
**Accuracy**	83.5%			89.6%			**91.2%**		

* Pre (precision), Sen. (Sensitivity), # indicates the amount of parameters, and bold indicates the best performance on this metric.

**Table 8:** the parameters and FlOPs of the model.

	Vgg16	Resnet50	Densenet121	Base Classifiers 1	Base Classifiers 2	Base Classifiers 3	COVIDx-LWNet
# *of Parameters	15 million	24 million	7 million	38 million	16 million	16 million	71 million
**FLOPs**	29 million	47 million	14 million	90 million	45 million	47 million	90 million

* # indicates the amount of parameters.

**Table 9:** D3_1:D3_1 (train: test), three-class.

	Vgg16	Resnet50	Densenet121	Base Classifiers 1	Base Classifiers 2	Base Classifiers 3	COVIDx-LWNet
**COVID_Sensitivity**	97.72	99.25	98.23	98.97	99.16	97.25	99.03
**COVID_specificity**	98.61	99.36	99.46	99.16	98.64	99.32	99.89
**Accuracy**	97.24	98.56	98.01	99.01	98.78	98.28	99.15

**Table 10:** D3_1:D1 (train: test), three-class.

	Vgg16	Resnet50	Densenet121	Base Classifiers 1	Base Classifiers 2	Base Classifiers 3	COVIDx-LWNet
**COVID_Sensitivity**	83.15	90.54	88.69	93.29	88.59	89.42	92.65
**COVID_specificity**	98.26	96.23	97.08	96.88	93.21	94.18	95.49
**Accuracy**	88.78	90.89	89.25	92.71	92.49	91.08	93.21

**Table 11:** D3_2:D3_2 (train: test), four-class.

	Vgg16	Resnet50	Densenet121	Base Classifiers 1	Base Classifiers 2	Base Classifiers 3	COVIDx-LWNet
**COVID_Sensitivity**	90.26	95.46	91.56	93.69	91.84	92.56	94.14
**COVID_specificity**	94.61	98.16	98.72	98.89	99.25	98.47	99.26
**Accuracy**	90.79	92.03	93.14	93.21	92.4	91.73	94.86

**Table 12:** D3_2:D2 (train: test), four-class.

	Vgg16	Resnet50	Densenet121	Base Classifiers 1	Base Classifiers 2	Base Classifiers 3	COVIDx-LWNet
**COVID_Sensitivity**	84.64	91.26	89.54	93.46	89.69	88.65	92.56
**COVID_specificity**	90.17	97.78	96.45	98.43	95.42	94.46	98.75
**Accuracy**	80.47	84.89	83.53	87.26	86.94	85.6	88.65

**Table 13:** Comparison of the proposed system with existing systems in terms of accuracy.

Year	Author	Classes	Type	Model	Accuracy
**2020**	Shibly et al. [32]	2 Class: (COVID: 183, normal: 13,617)	Chest X-ray	R–CNN	97.36%
**2020**	Tulin Ozturk et al. [33]	3 Class (COVID: 127, normal: 500, pneumonia: 500)	Chest X-ray	DarkCOVIDNet	87.02%
**2021**	Law and Lin. [34]	3 Class (COVID: 1200, normal: 1341, pneumonia: 1345)	Chest X- ray	VGG-16	94%
**2021**	Francis Jesmar P. Montalbo [35]	3 Class (COVID: 1281, normal: 3270, pneumonia: 4657)	Chest X- ray	Fused-DenseNet-Tiny	97.99%
**2022**	Abhijit Bhattacharyya et al. [36]	3 Class (COVID:342, normal: 341, pneumonia: 347)	Chest X- ray	VGG-19, BRISK and RF	96.60%
**2022**	Anubhav Sharma et al. [18]	4 Class (COVID: 305, normal: 375, pneumonia_bacterial: 355 pneumonia_viral: 379)	Chest X-ray	COVDC-Net	90.22%
**2022 (ours)**	Proposed model	3 Class (**D1**)	Chest X-ray	COVIDX-LwNet	3 class, D1: 95.56%
		4 Class (**D2**)			4 class, D2: 91.2%
		3 Class, 4 Class (**D3**)			3 class, D3: 99.15% 4 class, D3: 94.86%

## Data Availability

Given in Table 1 in the main text.
